# Microarray Analysis of Gene Expression Involved in Butyrate-Resistant Colorectal Carcinoma HCT116 Cells

**DOI:** 10.31557/APJCP.2020.21.6.1739

**Published:** 2020-06

**Authors:** Chakkraphong Khonthun, Nongluk Saikachain, Siam Popluechai, Kongkiat Kespechara, Art Hiranyakas, Metawee Srikummool, Damratsamon Surangkul

**Affiliations:** 1 *Department of Biochemistry, Faculty of Medical Science, Naresuan University, Phitsanulok, Thailand. *; 2 *School of Science, Mae Fah Luang University, Chaiang Rai, Thailand. *; 3 *Gut microbiome research group, Mae Fah Luang University, Chaiang Rai, Thailand. *; 4 *Bangkok Hospital Pattaya, Chonburi, Thailand. *; 5 *Bangkok Hospital Phuket, Phuket, Thailand. *

**Keywords:** Butyrate, chemoresistance, colorectal cancer, cancer stem cells

## Abstract

**Background:**

Resistance to chemotherapeutic agents is usually found in cancer stem cells (CSCs) and cancer stem-like cells that are often regarded as the target for cancer monitoring. However, the different patterns of their transcriptomic profiling is still unclear.

**Objective:**

This study aims to illustrate the transcriptomic profile of CSCs and butyrate-resistant colorectal carcinoma cells (BR-CRCs), by comparing them with parental colorectal cancer (CRC) cells in order to identify distinguishing transcription patterns of the CSCs and BR-CRCs.

**Methods:**

Parental CRC cells HCT116 (HCT116-PT) were cultured and induced to establish the butyrate resistant cell model (HCT116-BR). Commercial enriching of the HCT116-CSCs were grown in a tumorsphere suspension culture, which was followed firstly by the assessment of butyrate tolerance using MTT and PrestoBlue. Then their gene expression profiling was analyzed by microarray.

**Results:**

The results showed that both butyrate-resistant HCT116 cells (HCT116-BR) and HCT116-CSCs were more tolerant a butyrate effects than HCT116-PT cells. Differentially expressed gene profiles exhibited that *IFI27*, *FOXQ1*, *PRF1*, and *SLC2A3* genes were increasingly expressed in CSCs, and were dramatically overexpressed in HCT116-BR cells when compared with HCT116-PT cells. Moreover, *PKIB* and* LOC399959* were downregulated both in HCT116-CSCs and HCT116-BR cells.

**Conclusion:**

Our findings shed light on the transcriptomic profiles of chemoresistant CRC cells. This data should be useful for further study to provide guidelines for clinical prognosis to determine the guidelines for CRC treatment, especially in patients with chemoresistance and designing novel anti-neoplastic agents.

## Introduction

Colorectal cancer (CRC) is the most frequently diagnosed cancer (Rawla et al., 2019), but the critical obstacle in its treatment is still the recurrence of the tumor during long-term survival after treatment. Recurring tumors are endurable to radiation therapy (Tang et al., 2018) and chemotherapy (Zeng, 2017), and are associated with poor prognosis and ineffective clinical outcomes. Currently, cancer stem cells and cancer stem-like cells are focused on elucidating tumor recurrence and cancer therapeutic resistance (Koren and Fuchs, 2016). Cancer stem cells (CSCs) possess the stemness characteristics that are distinct from other cancerous cells, such as self-renewal, chemoresistance, and tumorigenic potential (Tirino et al., 2013). Chemoresistance in CSCs results from the expression of chemoresistance-related molecules, which includes ATP-binding cassette (ABC) transporter, aldehyde dehydrogenase (ALDH), pro-survival BCL-2 protein family members (Abdullah and Chow, 2013), oncogenes (Zheng, 2017). Moreover, aberration in CSC signaling, including AKT/PBK, WNT/β-Catenin, Notch, and Sonic hedgehog are associated with drug resistance (Abdullah and Chow, 2013).

Cancer stem-like cells are proposed by the clonal evolution model that describes genetic instability, which leads to chemoresistant differentiation through expression of chemoresistant molecules (Ibragimova et al., 2017; Klemba et al., 2018). Drug-insensitive CRC cells often possess tumorgenic potential. Several studies demonstrated that acquired resistance to 5-FU (Liu et al., 2014), docetaxel (EI Khoury et al., 2016), oxaliplatin (Yan et al., 2017), and butyrate (Serpa et al., 2010) related to tumor formation *in vivo*. 

Butyrate, the histone deacetylase inhibitor (HDACi), is derived from dietary fibre and resistant starch formed by bacterial fermentation in the colon, and acts as an energy source for colonocytes and plays a role in colonic homeostasis. Like other HDAC inhibitors, it inhibits cell proliferation (Zeng et al., 2017) and induces apoptosis in cancerous cells (Ruemmele et al., 2003; Pajak et al., 2007; Roy et al., 2009). It has been suggested as the anti-proliferating agent with slight side effects for CRC treatment, and chronic exposure of it causes butyrate insensitivity (Kang et al., 2016). It has also been demonstrated that butyrate-rich colonic microenvironment contributes to tumor aggressiveness, resulting from the adaptation of subpopulation of butyrate-challenging CRCs (Serpa et al., 2010).

CSCs and butyrate-resistant colorectal carcinoma cells (BR-CRCs) are insensitive to chemotherapeutic drugs, while still being barriers to CRC treatment. In butyrate-rich condition, it seems that BR-CRCs express cancer stem-like features, and it has been reported that acquired butyrate resistance also overcomes anticancer effects of other chemotherapeutic drugs, such as paclitaxel, 5-fluorouracil, doxorubicin and trichostatin A (Kang et al., 2016). The *in vivo* studies demonstrated that butyrate resistance related to tumorigenicity of the CRC cells, were associated with aggressive phenotypes (Serpa et al., 2010). Although they are tolerant to antineoplastic agents, their gene expression profiles are still not fully understood. In this study, we established a BR-CRCs model from colorectal carcinoma cells, and the transcriptome profiling was assessed by comparing differentially expressed genes of CSCs, BR-CRCs, and CRCs. The profiles of the gene expressions will provide an insight to the transcriptomic patterns and candidate genes that relates to drug resistance for monitoring the CRC treatment and effective therapy.

## Materials and Methods

*Cell culture*

HCT116 colon cancer cell lines were obtained from American Type Culture Collection (ATCC). The cells were grown as monolayer in McCoy’s 5A with L-glutamine (Corning, USA) supplemented with 10% heat inactivated fetal bovine serum (HI-FBS) and 100U/ml penicillin, 100 μg/ml streptomycin and 0.25 μg/ml amphotericin B, then cultured at 37ºC in a humidified atmosphere of 95% air and 5% CO_2_. 

The parental HCT116 (HCT116-PT) cells were cultured in complete media, with a gradually increasing concentration of sodium butyrate (NaBu) for each 2 passages (Fung et al., 2009) by adding of 0.5-3.0 mM NaBu. Finally, the surviving cells were maintained with a media containing 3 mM NaBu to produce a butyrate-resistant HCT116 cell model (HCT116-BR).

The HCT116-CSCs were purchased from Promab Biotechnologies (Promab Biotechnologies, USA). Tumorspheres were grown in an ultra-low binding plate and cultured with Cancer Stem Premium™ media (Promab Biotechnologies, U.S.A.) containing 100U/ml penicillin, 100 μg/ml streptomycin and 0.25 μg/ml amphotericin B. CSCs aggregated to form the tumorsphere which were visible by an inverted light microscope, followed by subcultivation that was performed every 8-10 day. 

*Assessment of butyrate tolerance by MTT and PrestoBlue assay*

To confirm the butyrate-resistant ability, cell viability was assessed by MTT reduction assay. HCT116-PT and HCT116-BR cells were seeded in a 96-well plate with a density of 5x10^3^ cells/well, and incubated for sufficient attachment before NaBu treatment. The HCT116-PT cells were treated with various concentrations of 0, 1, 3, 5, 7, 10 mM NaBu (Sigma Aldrich, Switzerland) for 24 and 48 h. McCoy’s 5A containing 3 mM NaBu was used for HCT116-BR cell seeding followed by NaBu treatments, while the control group was exposed to 3 mM NaBu for the same incubation period as the HCT116-PT cells, 1 mg MTT (Ameresco, USA) was added to each well, and was further incubated for 4 h under a culture conditions. Purple formazan crystals were determined using a spectrophotometrically at 540 nm. The PrestoBlue assay was used to assess the butyrate tolerance of HCT116-CSCs. The cells were seeded in a 96-well plate with a density of 5x10^3^ cells/well and treated with the same NaBu concentrations and incubation period. The PrestoBlue™ Reagent (Invitrogen, USA) was added into the culture media and incubated for 2 h, then the fluorescence was measured using a microplate reader (BioTek, USA) at an excitation of 535 and an emission of 615 nm.

*Microarray analysis*

The expression of human genes was analyzed using a microarray analysis to simultaneously assess the mRNA expression. Cell samples of HCT116-PT, HCT116-BR cells, and HCT116-CSCs were sent to Macrogen, Inc (Macrogen, South Korea). Global gene expression profiling was assessed by using microarray analyzer (Illumina, USA). Total RNA was amplified and purified using TargetAmp-Nano Labeling Kit for Illumina Expression BeadChip (EPICENTRE Biotechnologies, USA) to yield biotinylated cRNA, 200 ng of the total RNA was reverse-transcribed to cDNA using a T7 oligo(dT) primer and labeled with biotin-NTP. Labeled cDNA were hybridized to each Human HT-12 v4.0 Expression Beadchip (Illumina, USA). Signals were detected by using Amersham fluorolink streptavidin-Cy3 (GE Healthcare Bio-Sciences, UK). Arrays were scanned with an Illumina bead array Reader confocal scanner. Raw data were extracted using the software provided by the manufacturer (Illumina GenomeStudio v2011.1 (Gene Expression Module v1.9.0)). The fold change of gene expressions was considered in HCT116-CSCs vs HCT116-PT cells and HCT116-BR vs HCT116-PT cells. Gene selection was based on the top 10 rankings of the increased fold changes of gene expressions in HCT116-CSCs and HCT116-BR cells when compared with HCT116-PT cells that clearly indicated their genetic features.

*Data analysis*

Cell viability values are represented as mean ± S.M.E, while statistical differences between the two groups were determined with the Student’s t test. Significance is when the *p* value is less than 0.05. Data form the microarray was exhibited as the relative gene expression of HCT116-CSCs vs HCT116-PT cells and HCT116-BR vs HCT116-PT cells, which were listed in descending order to study their transcriptomic profiling.

## Results

*The effect of NaBu on the cell viability of HCT116-CSCs, HCT116-BR, and HCT116-PT cells*

**Figure 1:**
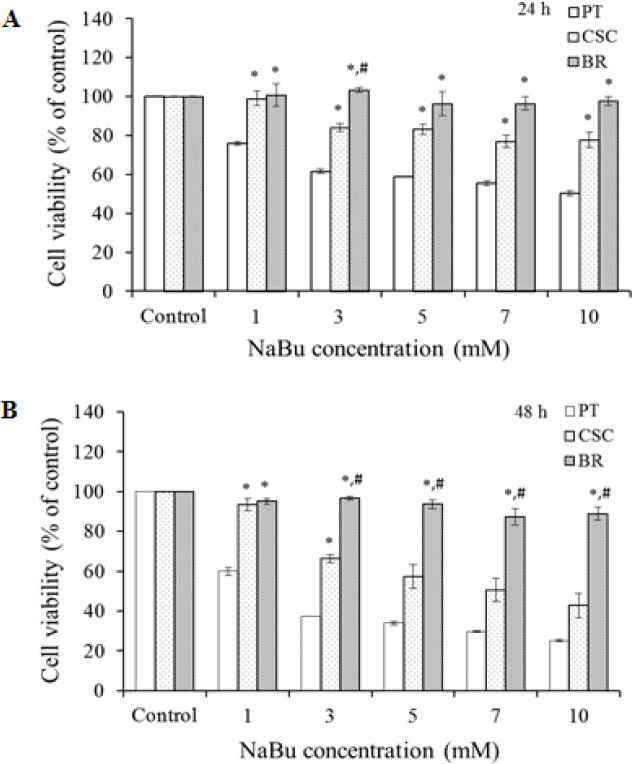
The Effect of NaBu on the Cell Viability of HCT116-PT, HCT116-BR Cells and HCT116-CSCs. The cells were treated with different concentrations of NaBu (0, 1, 3, 5, 7, and 10 mM) for 24 (A) and 48 h (B), followed by the cell viability being evaluated using an MTT and PrestoBlue assay then presented as a percentage of control. (*p*<0.05 *significance with HCT116-CSCs and HCT116-PT cells, ^#^significance with HCT116-BR cells and HCT116-CSCs).

To assess the effect of NaBu on cell viability, HCT116-PT, HCT116-BR cells, and CSCs were treated with different concentrations of NaBu (0, 1, 3, 5, 7, and 10 mM) for 24 and 48 h. The survival of the HCT116-PT and HCT116-BR cells were evaluated by an MTT reduction assay, and Butyrate tolerance of CSCs was investigated using the PrestoBlue assay. The results showed that NaBu decreased cell viability of the HCT116-PT cells and CSCs in concentration and a time-dependent manner, while the endurance of butyrate increased in the HCT116-BR cells (Figure 1A and 1B).

*Different gene expression of HCT116-PT/CSCs and HCT116-BR/HCT116-PT cells*

Transcriptomic profiling was obtained by microarray analysis. The total RNA of the HCT116-PT, HCT116-BR cells, and HCT116-CSCs were reverse-transcribed to cDNA and subsequently hybridized to each Human HT-12 v4.0 Expression Beadchip (Illumina, Inc., San Diego, USA). The levels of gene expression were calculated and represented as a fold change of expression of HCT116-CSCs vs HCT116-PT cells and HCT116-BR cells vs HCT116-PT cells. The values indicated up- or down-regulation of genes between them. For preliminary screening, 10 upregulated and 10 downregulated genes were ranked in descending order.

*Differentially expressed genes of HCT116-CSCs, compared with the HCT116-PT cells*

The comparison of global gene expression between HCT116-CSCs and HCT116-PT cells was conducted to assess the gene expression profiling that indicated the intrinsic resistance to butyrate and cancer stemness. The microarray data revealed that the top 10 up-regulated genes that mostly participated in aggressive phenotype and cancer progression, consisting of *CEACAM1, IFI27, FOXQ1, PRF1, SNTB1, FGFBP1, ZBED2, H19, FSTL5*, and *SLC2A3* genes. These could be classified into 3 groups, (1) the metastasis and tumorigenesis-promoting factors containing *CEACAM1, IFI27, FOXQ1*, and H19, (2) survival molecules; FGFBP1 and SLC2A3, and (3) tumor-associated factors, including* PRF1, SNTB1, ZBED2, *and *FSTL5.* The cancer stemness features were associated with slightly decreased expression of *PKIB, TRNP1, RGS2, STK39, CYR61, MMP7,* and *PPARG* genes which often participated in cell proliferation (Table 1).

*Differentially expressed genes of the HCT116-BR cells, compared with the HCT116-PT cells*

HCT116-BR cells which are more resistant to butyrate effects exhibited the top 10 upregulations of* IFI27, BST2, SLC2A3, KRT13, DHRS2, IFI6, PRF1, PLAC8, OLFM1, *and *FOXQ1* genes. They could be classified as (1) tumor growth and metastasis promoting factors containing *IFI27, FOXQ1, KRT13, IFI6, *and *PLAC8* genes, (2) glucose transporter has the *SLC2A3* gene, (3) the chemoresistance-associated molecules comprising the *DHRS2* and *BST2* genes, and (4) the *PRF1* and *OLFM1* genes with unclear functions. Interestingly, *IFI27*, *FOXQ1*, and *SLC2A3* genes were dramatically overexpressed when compared with parental cells. The 10 downregulated genes were *LOC399959, MKX, ARMC4, ACSL5, GPR110, SCG2, PKIB, NT5E*, and *AKAP12* which were frequently found in several cancers, but their roles remain unclear (Table 2).

*The gene expression in both HCT116-CSCs and HCT116-BR cells*

**Figure 2:**
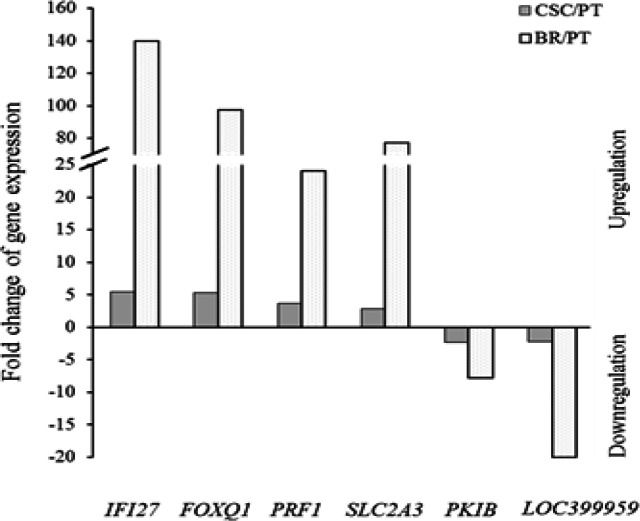
A Fold Changes of the Differentially Expressed Genes in Both HCT116-CSCs and HCT116-BR Cells, compared with the HCT116-PT cells. IFI27, FOXQ1, PRF1, and SLC2A3 genes were upregulated, while PKIB and LOC39959 genes were downregulated by varying degree

According to microarray data, the gene expression profiling showed 4 upregulated genes and 2 downregulated genes that were found in both the HCT116-CSCs and the HCT116-BR cells by varying degree (Figure 2). The upregulations of *IFI27*, *FOXQ1*,* SLC2A3*, and *PRF1* genes in the HCT116-CSCs were detected at 5.42, 5.38, 3.66, and 2.83 folds, respectively whereas extensive overexpressions by 139.86, 97.37, 77.21, and 24.12 folds, respectively were found in the HCT116-BR cells (Table 1). The downregulated genes, *PKIB* and* LOC399959*, were slightly decreased in HCT116-CSCs (Table 1) by approximately 2 fold, while expressions of *LOC399959* and* PKIB *were apparently decreased more than 19 and 7 folds in HCT116-BR cells when compared with HCT116-PT cells (Table 2). These results revealed the correlation of upregulated and downregulated genes which are implicated in intrinsic and acquired resistance of CSCs and BR cells, respectively.

## Discussion

Tumor recurrence and therapeutic resistance are considered as clinical problems for CRC treatment. The reappearance of tumors leads to poor prognosis and ineffective clinical outcomes. The subpopulation of chemoresistant CRC cells that are distinct from cancer cells in bulky tumors, are often referred to as CSCs and cancer stem-like cells that upregulate the chemoresistance-related molecules by providing drug-fighting mechanisms and contributing to therapeutic failure (Abdullah and Chow, 2013; Murayama and Gotoh, 2019). Butyrate, the anti-neoplastic agent, turns on several anti-cancer mechanisms (Zeng et al., 2017), however, long-term exposure to butyrate mediates the acquisition of resistance to chemotherapeutic drugs (Kang et al., 2016). This study established the butyrate-resistant cell model to reveal the transcriptomic profiles of chemoresistant cells. Our results exhibited that exposure to butyrate caused a decrease in the cell viability of HCT116-PT cells, both in concentration and time-dependent manners, however, our studies found that HCT116-CSCs and HCT116-BR cells were more resistant to the butyrate effects than HCT116-PT cells (Figure 1A and 1B). The intrinsic chemoresistance exist in CSCs (Koren and Fuchs, 2016) whereas acquired chemoresistance is induced with chronic activation with chemotherapeutic drugs. It has been proposed that resistance to butyrate associated with cancer progression (Lazarova and Bordonaro, 2016), and butyrate-rich colonic microenvironment contributes to tumor aggressiveness resulting from the adaptation of subpopulation of butyrate-challenging CRC cells in animal model (Serpa et al., 2010). The assessment of the gene transcription profiles of HCT116-CSCs/HCT116-PT cells and HCT116-BR/HCT116-PT cells revealed that expressions of *IFI27, FOXQ1, PRF1*, and *SLC2A3 *genes were highly increased in HCT116-CSCs, and being dramatically overexpressed in the HCT116-BR cells when compared with the HCT116-PT cells. The analysis of gene expression data also showed downregulations of *PKIB* and *LOC399959* that their levels were slightly decreased in HCT116-CSCs, and greatly declined in HCT116-BR cells (Table 1 and 2). 

A slight increase of *IFI27*, *FOXQ1*, *PRF1*, and *SLC2A3* genes in CSCs probably implicate in phenotypes of cancer stem cells that were often quiescent, but being able to exhibit their chemoresistant properties. However, overexpressions of these genes in *HCT116-BR* cells might be resulted from the establishing of defensive mechanisms against butyrate-induced apoptosis, reflecting cell adaptation responsible for butyrate stress. 

Interferon inducible factor27 (IFI27), a hydrophobic mitochondrial protein, has been reported in psoriatic skin (Suomela et al., 2004) and several cancer cells with aggressive phenotypes (Budhu et al., 2007; Li et al., 2015; Wang et al., 2018; Chiang et al., 2019). IFI27 not only induced ovarian tumorigenicity, stemness, but also participated in drug resistance of ovarian cancer cells (Li et al., 2015). Deregulation of* IFI27* expression has been reported in cholangiocarcinoma patients that are associated with poor survival (Chiang et al., 2019). Elevated expression of *IFI27* in HCT116-CSCs might implicate in their cancer stemness characteristics, particularly tumorigenicity. Our results revealed that long-term exposure to butyrate not only established a tolerance to butyrate, but also caused an excess *IFI27* mRNA expression in HCT116-BR cells. Studies by Virag and colleagues (2013) demonstrated that transcriptional activity for* IFI27* gene was increased by acquired resistance to oxaliplatin in HT-29R cells. Currently, it is well known that the epithelial-mesenchymal transition (EMT) is linked to chemoresistance (Elaskalani et al., 2017; Staalduinen et al., 2018) and cancer progression (Ramos et al., 2017). Study by Li et al (2015) demonstrated that IFI27 promoted EMT and drug resistance. Therefore, excess expression of *IFI27* in HCT116-BR cells might be the defensive mechanisms responsible for chronic butyrate activation and mainly implicated in aggressive phenotypes.

FOXQ1, a transcription factor, acted as the key player in the pathogenesis of tumors (Li et al., 2016) and correlated with poor prognosis (Zhan et al., 2015). It played a role in EMT in human cancer (Qiao et al., 2011), and has been reported that an upregulation of it was found in the metastatic stage of breast cancer (Zhang et al., 2011), esophageal cancer (Pei et al., 2015), pancreatic cancer (Li et al., 2016) and gastric cancer (Guo et al., 2017). In colorectal cancer, FOXQ1 not only promoted metastatic potential (Abba et al., 2013; Liu et al., 2017) but also enhanced tumorigenicity and tumor growth (Keneda et al., 2010). Elevated expression of *FOXQ1* in HCT116-CSCs may be involved in regulation of expression pattern of genes which determine the characteristics of cancer stem cells. Excess* FOXQ1 *transcription was clearly found in HCT116-BR cells that might participated in gene regulation of butyrate resistance pattern and responding to butyrate actions, especially cell differentiation, and the induction of defensive mechanisms against the butyrate-induced apoptosis, and the HCT116-BR cells may also have metastatic potential that require further investigation. 

The accelerated glycolytic activity is the hallmark of cancer that is associated with high glucose consumption by glucose transporters (GLUTs). Our results revealed that expression of the* SLC2A3* gene, encoding for GLUT3 protein, was raised in HCT116-CSCs. Christensen et al., (2015) reported that expression of it correlated with OCT4, which is a candidate marker for CSCs and the NANOG and is regulated by genes which participate in the embryonic and cancer stem cell program (Korkola et al., 2006). CSCs reside in a tumor mass where they often encounter hypoxic conditions and glucose deprivation, therefore, it is possible that the SLC2A3 upregulation may respond to the microenvironment of CSCs. The acquisition of resistance to butyrate caused overexpression of *SLC2A3* mRNA 77.21 fold that might denote the mechanisms responsible for cell survival, especially chemoresistance, because ATP are necessary for the activity of ATP-binding cassette (ABC) transporters which extrude chemotherapeutic agents. Previous studies showed the correlation between chemoresistance and *SLC2A1* (Song et al., 2014; Wang et al., 2017). However, overexpression of *SLC2A3* and drug resistance is less well defined. GLUT3 served as the transporter with high affinity responsible for rapid glucose uptake. It is possible that enhanced *SLC2A3* expression in HCT116-BR cells were defensive mechanisms, responding to butyrate stress, and ultimately contributed to aggressive phenotypes.

Perforin-1 (PRF1) which is normally found in the granules of cytotoxic T lymphocytes (CTLs) and natural killer cells, plays a role in immune response (Podack and Munson, 2016). Our results revealed that its upregulation could be detected both in HCT116-CSCs and HCT116-BR cells, while its role in cancer and chemoresistance has not yet been identified. 

Our results showed the decreased expression of *PKIB* and* LOC399959* in both HCT116-CSCs and HCT116-BR cells. PKIB promoted cell proliferation (Dou et al., 2016), while slight down-regulation of it appeared in the HCT116-CSCs which might participate in the feature of cancer stem cells, but their expression greatly reduced in the HCT116-BR cells It is possible that a decrease in the *PKIB* expression is a strategy to reduce cell activity and preserve energy for the survival of CSCs and the downregulation in HCT116-BR cells, may decrease in response to butyrate-induced apoptosis, which leads to butyrate resistance, while LOC399959 is a non-coding RNA, whose function in cancer is still unknown. The previous studies focused on butyrate resistance in adenocarcinoma by using HT29 (Fung et al., 2009) and BCS-TC2 cells (Silanes et al., 2004), however, HCT116-BR cells have been reported by Kang et al., (2016). Who established the butyrate resistant CRC and maintained in media containing 1.6 mM butyrate which differed our study. They demonstrated that acquired resistance to butyrate led to chemoresistance and did not point to gene expression. Our BR cell model that was induced with 0.5-3.0 mM and maintained with 3.0 mM butyrate has never been found elsewhere.

In conclusion, chemoresistant cells are referred to as CSCs and cancer stem-like cells, so even though they are not identical, CRC treatment is focused on eradicating both of them. The upregulations of the *IFI27*, *FOXQ1*, *PRF1*, and *SLC2A3* genes and the downregulation of the *LOC399959* and *PKIB* genes by varying degrees, possibly implicate the resistance to butyrate of the HCT116-CSCs and the HCT116-BR cells. A slight change in the gene expression of the HT116-CSCs may be involved in the CSC niche and chemoresistance, whereas overexpression in the HCT116-BR cells, might result from butyrate-induced cell adaptation or the defensive mechanisms responsible for chronic butyrate activation that may predominantly implicate the aggressive phenotypes. Although transcriptomic patterns of intrinsic and acquired resistance to butyrate are not identical, we expected that either high or overexpression of these genes were useful in selecting and designing novel anti-cancer agents for the effective targeting of CRC therapy.

**Table 1:** Differentially Expressed Gene Profiles of HCT116-CSCs/HCT116-PT cells

Gene symbol	Gene ID	Relative expression	Definition
Upregulated genes			
* CEACAM1*	634	5.611914	Carcinoembryonic antigen-related cell adhesion molecule 1
* IFI27*	3429	5.421592	Interferon alpha-inducible protein 27
* FOXQ1*	94234	5.378125	Forkhead box Q1
* PRF1*	5551	3.659196	Perforin-1
* SNTB1*	6641	3.631548	Syntrophin Beta 1
* FGFBP1*	9982	3.210627	Fibroblast growth factor binding protein 1
* ZBED2*	79413	3.147975	Zinc finger BED-type containing 2
* H19*	283120	3.101951	non-coding RNA
* FSTL5*	56884	2.991059	Follistatin like 5
* SLC2A3*	6515	2.826233	Facilitated glucose transporter member 3
Downregulated genes			
* NR4A3*	8013	2.544763	Nuclear receptor subfamily 4 group A member 3
* PKIB*	5570	2.312368	Protein kinase (cAMP-dependent, catalytic) inhibitor beta
* TRNP1*	388610	2.311719	TMF1-Regulated Nuclear Protein
* RGS2*	5997	2.192854	Regulator of G-Protein Signaling
* LOC399959*	399959	2.191778	Homo sapiens hypothetical LOC399959, non-coding
* STK39*	27347	2.138326	Serine/Threonine Kinase 39
* CYR61*	3491	2.084594	Cysteine Rich Angiogenic Inducer 61
* FER1L4*	80307	2.057106	Fer-1 like family member 4
* MMP7*	4316	2.04973	Matrix metallopeptidase 7
* PPARG*	5468	2.039141	Peroxisome proliferator activated receptor gamma

**Table 2:** Differentially Expressed Genes of HCT116-BR and HCT116-PT Cells

Gene symbol	Gene ID	Relative expression	Definition
Upregulated genes			
* IFI27*	3429	139.858	Interferon alpha inducible protein 27
* FOXQ1*	94234	97.373	Forkhead box Q1
* SLC2A3*	6515	77.212	Facilitated glucose transporter member 3
* KRT13*	3860	43.522	Keratin 13
* DHRS2*	10202	39.526	Dehydrogenase/reductase SDR family member 2
* IFI6*	2537	26.686	Interferon alphainducible protein 6
* PRF1*	5551	24.12	Perforin-1
* PLAC8*	51316	22.238	Placenta Specific 8
* OLFM1*	10439	19.194	Olfactomedin 1
* BST2*	684	19.128	Bone Marrow Stromal Cell Antigen 2
Downregulated genes			
* LOC399959*	399959	19.8651	non-coding RNA
* MKX*	283078	11.491	Mohawk homeobox
* ARMC4*	55130	10.6548	Armadillo repeat containing 4
* ACSL5*	51703	10.5275	Acyl-CoA synthetase long-chain family member 5
* GPR110*	266977	9.722	Adhesion G protein-coupled receptor F1 (ADGRF1)
* SCG2*	7857	8.21684	Secretogranin II
* PKIB*	5570	7.693	Protein kinase (cAMP-dependent, catalytic) inhibitor beta
* TFPI*	7035	7.08242	Tissue factor pathway inhibitor
* NT5E*	4907	6.84268	5'-nucleotidase ecto
* AKAP12*	9590	6.71525	A-Kinase Anchoring Protein 12
